# Eco‐Friendly ZnO–Chitosan Nanocomposite‐Embedded Sodium Alginate/Polyvinyl Alcohol/Saffron Hydrogel for Enhanced Antibacterial Applications

**DOI:** 10.1049/nbt2/7756674

**Published:** 2026-05-06

**Authors:** Fatemeh Karampour, Saba Zendehcheshm

**Affiliations:** ^1^ Department of Chemistry, National University of Skills (NUS), Tehran, Iran, nus.ac.ir; ^2^ Inorganic Chemistry Department, Faculty of Chemistry, Razi University, Kermanshah, Iran, razi.ac.ir

**Keywords:** antibacterial, chitosan, green synthesis, hydrogel, zinc oxide nanoparticle

## Abstract

This study reports the green synthesis, development, and characterization of novel eco‐friendly zinc oxide nanoparticles (ZnO NPs) coated with chitosan (ZnO NPs–Chsn) and their incorporation into a polyvinyl alcohol/sodium alginate/saffron (PSS) hydrogel for enhanced antibacterial applications. ZnO NPs were biosynthesized using quince (*Cydonia oblonga*) peel extract, yielding semi‐spherical particles with an average size of ~72 nm. The Chsn coating improved particle dispersion, surface uniformity, and colloidal stability while modifying the surface charge (zeta potential from −27 to −11.6 mV), thereby enhancing biocompatibility and antibacterial potential. The ZnO NPs–Chsn were integrated into the PSS hydrogel matrix via a freeze–thaw method to improve biocompatibility and generate a highly porous structure. Incorporation of ZnO NPs–Chsn increased the maximum swelling ratio from 220.91% ± 1.89% (PSS) to 589.39% ± 1.77% and enhanced hydrophilicity, as confirmed by contact angle (CA) measurements. Antibacterial assays (disc diffusion) showed that the PSS/ZnO NPs–Chsn hydrogel produced inhibition zones of 4.64 ± 0.20 mm (*S. aureus*), 1.55 ± 0.10 mm (*E. coli*), and 4.94 ± 0.14 mm (*B. cereus*), compared with negligible inhibition for the base PSS hydrogel. The enhanced antibacterial effect is attributed to the synergistic action of ZnO NPs and Chsn through bacterial membrane disruption, reactive oxygen species (ROS) generation, and metabolic impairment, potentially boosted by the bioactive compounds in saffron (crocin and safranal). These findings highlight the potential of PSS/ZnO NPs–Chsn hydrogels as eco‐friendly antibacterial biomaterials; however, further evaluations are required to confirm clinical applicability.

## 1. Introduction

The increasing global concerns regarding bacterial resistance to antibiotics and the limitations of conventional antimicrobial treatments have accelerated the pursuit of innovative strategies to combat bacterial infections [1]. Among the most promising solutions, hydrogels embedded with nanoparticles (NPs) have attracted significant attention due to their capability to provide prolonged antimicrobial activity, controlled drug release, and enhanced wound healing [2]. These nanocomposite hydrogels can be classified based on the type of NPs incorporated, including metal NPs, metal oxide NPs, and polymeric NPs, each offering distinct advantages in terms of antimicrobial efficacy and drug delivery [3–5]. Traditionally, NP synthesis has often relied on hazardous chemicals and solvents, posing risks to both human health and the environment. Green synthesis techniques provide a more sustainable and environmentally friendly alternative for NP production. These methods utilize natural and renewable resources as catalysts while minimizing the use of toxic substances. One widely adopted approach for eco‐friendly NP synthesis involves the use of plant extracts, which contain a variety of biologically active compounds that function as both reducing and stabilizing agents during NP formation [6, 7]. Additionally, microorganisms and enzymes can facilitate green NPs synthesis. Bacteria, fungi, and yeast have the capability to generate NPs by reducing metal ions, while enzymes such as lipases and proteases can also act as reducing agents in NP production. Among various biological sources, plants are recognized as highly effective for NP synthesis. Their higher tolerance to metal toxicity compared to bacteria and algae makes them particularly suitable for producing zinc oxide nanoparticles (ZnO NPs). In summary, the green synthesis of NPs presents a more sustainable and eco‐conscious method for their production [8]. The quince fruit (*Cydonia oblonga*, Rosaceae) is native to western Asia, particularly Iran to Turkestan, and is now cultivated worldwide, including Europe, Oceania, South America, Australia, Africa, and parts of Asia [9, 10]. Quince is rich in vitamins (B_1_, B_2_, PP, and C), organic acids (malic and citric), carbohydrates (starch, fructose, and glucose), carotene, tannins, aromatic compounds, fiber, minerals (Mg, P, K, and Ca), proteins, and antioxidants like caffeoylquinic acids and flavonoids, including quercetin 3‐galactoside, kaempferol‐3‐rutinoside, and kaempferol 3‐glucoside [11]. These compounds contribute to nanosystem formation and surface modification, with polyphenols in the peel, leaves, and seeds playing a key antioxidant role [12, 13].

Hydrogels are highly valued for their significant water content, biocompatibility, and biodegradability, making them particularly suitable for biomedical applications, especially in wound healing and infection management. Integrating NPs such as antibacterial metallic NPs, polymeric NPs, and NPs loaded with antimicrobial agents into hydrogel matrices has been shown to greatly improve their antimicrobial efficacy, mechanical properties, and ability to support cell proliferation. Among these, metal NPs, including silver (Ag), gold (Au), copper (Cu), and ZnO, exhibit broad‐spectrum antimicrobial properties, positioning them as effective alternatives to conventional antibiotics. Notably, ZnO NPs stand out due to their strong antimicrobial activity, minimal cytotoxicity, and capacity to target a wide range of pathogens through mechanisms such as ion release and the generation of reactive oxygen species (ROS). Moreover, hydrogels embedded with ZnO NPs have demonstrated effectiveness in treating infections caused by both Gram‐positive and Gram‐negative bacteria, as well as fungi [14–17]. Polymeric NPs, such as Chsn and poly(lactic‐co‐glycolic acid) (PLGA), have been extensively investigated for their antimicrobial properties. When integrated into hydrogels, these NPs not only boost the antimicrobial effectiveness but also create a platform for the controlled release of antimicrobial agents, leading to sustained therapeutic effects with reduced dosing frequency. Additionally, polymeric NPs can act as carriers for other therapeutic substances, such as antibiotics and peptides, thereby further enhancing the antimicrobial potential of the hydrogel system [18–20]. The potential to modify hydrogels by incorporating active ingredients, such as plant extracts, offers new opportunities in the realm of topical treatments and controlled drug delivery [21]. The bioactive chemical compounds most extensively studied in biological extracts include polyphenols, flavonoids, alkaloids, tannins, and terpenoids, which possess a wide array of biological activities, such as antioxidant, anti‐inflammatory, and antimicrobial properties [22]. Since biological extracts are primarily of natural origin, they are considered safe and are increasingly used as alternatives to chemical antibiotics for antimicrobial activities.

Saffron (*Crocus sativus L*.), a perennial plant from the Iridaceae family, is mainly cultivated in Iran, India, Morocco, Greece, Spain, and Italy [23, 24]. It possesses pharmacological properties such as antispasmodic, expectorant, antibacterial, antifungal, antioxidant, anti‐inflammatory, antihypertensive, hypolipidemic, antidepressant, and antitumor effects. Its antimicrobial activity is linked to compounds like safranal and crocin. The petals also contain flavonoids, anthocyanins, vitamins (riboflavin and thiamin), proteins, minerals, and gums [25]. The antimicrobial potential of saffron is mainly attributed to compounds such as crocin, safranal, and phenolics. These compounds can disrupt bacterial cell membranes, interfere with cellular protein functions, reduce membrane permeability, and chelate essential metal ions, thereby impairing microbial metabolic activity. Through these multiple simultaneous mechanisms, saffron effectively inhibits microbial growth [26]. Saffron, with its active compounds such as crocin, safranal, and flavonoids, accelerates wound healing by reducing inflammation, enhancing antioxidant activity, stimulating collagen synthesis, and promoting angiogenesis. Studies have shown that saffron petal extract increases the proliferation and migration of fibroblasts and endothelial cells, improves extracellular matrix deposition, and enhances the formation of new blood vessels, ultimately leading to faster and more organized wound closure, particularly in chronic and diabetic wounds [27].

In this study, a hydrogel based on PVA and SA was prepared, and to enhance its antimicrobial performance, saffron extract and green‐synthesized ZnO NPs from quince (*Cydonia oblonga*) peel coated with Chsn (ZnO NPs–Chsn) were incorporated. Active compounds in saffron, such as crocin, safranal, and phenolics, can exert synergistic effects with ZnO NPs–Chsn by disrupting bacterial cell membranes, interfering with essential proteins and enzymes, reducing membrane permeability, and chelating vital metal ions, thereby enhancing the hydrogel’s antimicrobial activity. Furthermore, due to saffron’s antioxidant and anti‐inflammatory properties, the potential wound‐healing effects of this hydrogel can be explored in future studies.

## 2. Materials and Methods

### 2.1. Preparation of the Quince Fruit (*Cydonia oblonga*) Peel Extract

**Figure 1:**
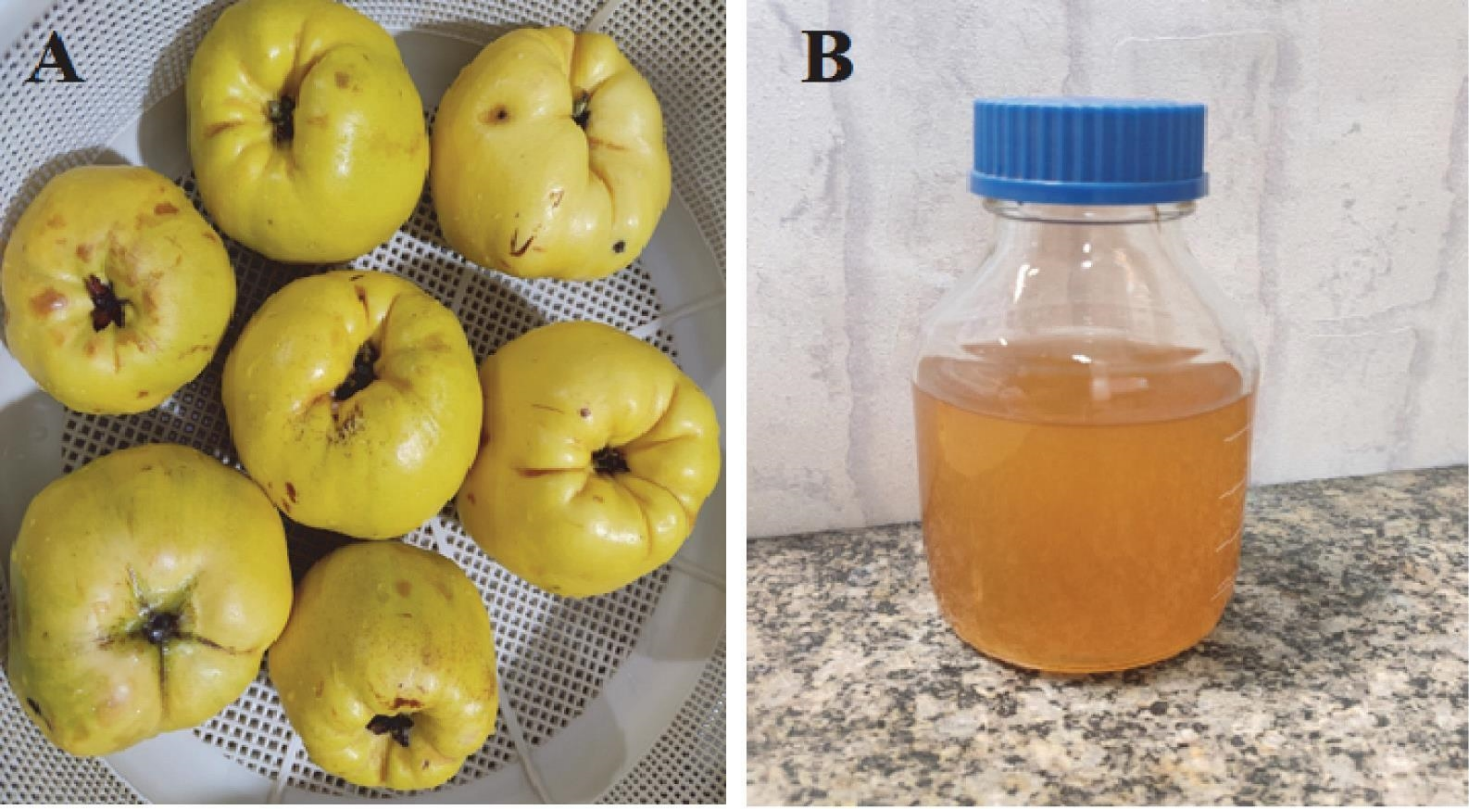
(A) Fresh quince fruits (*Cydonia oblonga*) and (B) aqueous quince extract after filtration, used for green synthesis of nanoparticles.

Quince fruits (*Cydonia oblonga*) were obtained (Figure 1A), and their peels were carefully separated. The peels were thoroughly washed with distilled water to remove any impurities and then air‐dried in the shade for 5–7 days. Once completely dried, they were ground into a fine powder. About 10 g of the powdered peels were added to 100 mL of deionized water and heated to 80°C for 30 min to obtain a concentrated yellowish‐brown extract. After cooling to room temperature, the extract was filtered using Whatman filter paper (pore size 41) and stored for further use (Figure 1B).

### 2.2. Green Synthesis of ZnO NPs

**Figure 2:**
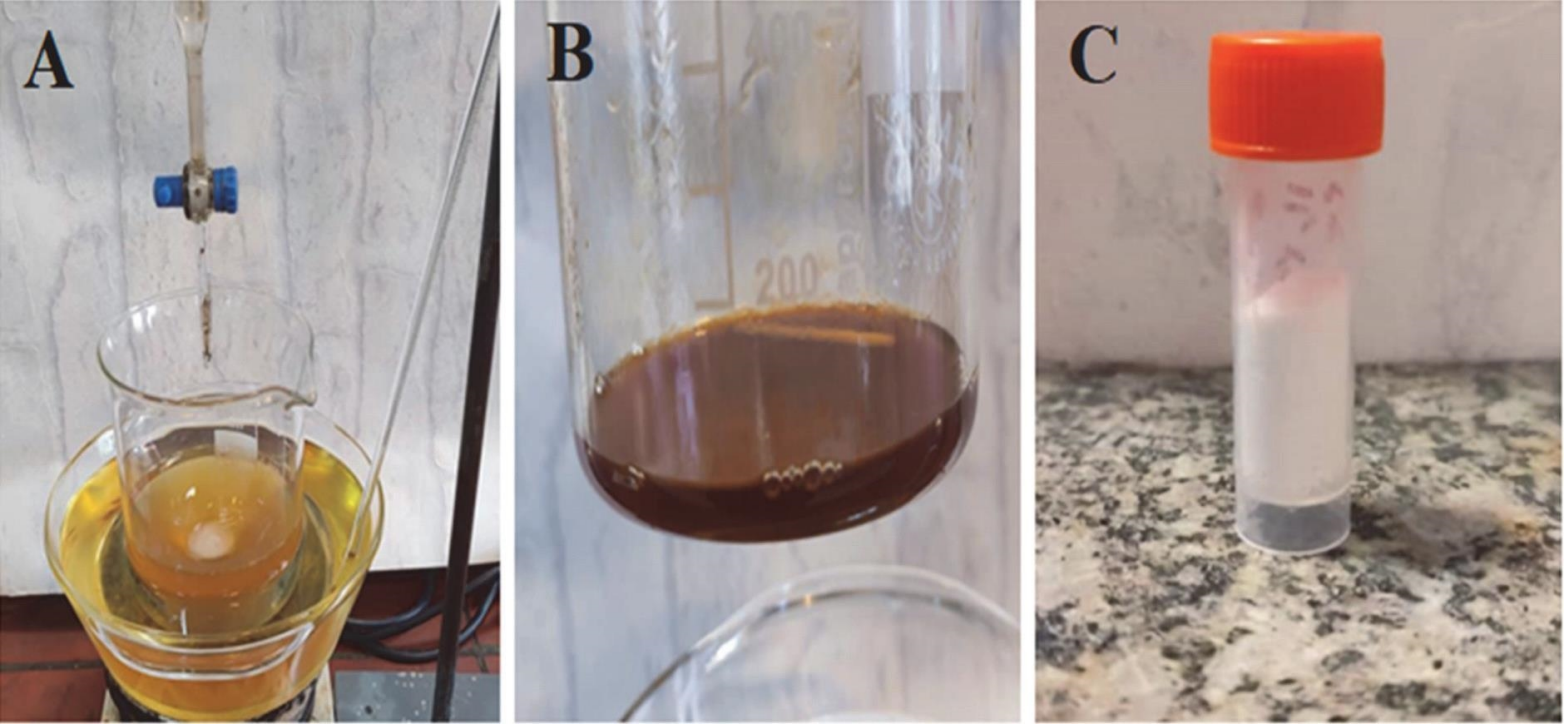
Green synthesis process of ZnO NPs. (A) Dropwise addition of 0.1 M zinc nitrate solution into preheated quince extract at 40°C, followed by stirring at 80°C for 2 h. (B) Concentration of the mixture by heating to 90°C, forming a dark gel. (C) Final white ZnO nanopowder obtained after drying and calcination at 700°C for 2 h.

The ZnO NPs were synthesized following this method: a 100 mL solution of zinc nitrate (Zn(NO_3_)_2_·6H_2_O, 0.1 M) was gradually added drop by drop into 100 mL of the extract, which had been preheated to 40°C (Figure 2A). The mixture was continuously stirred at 80°C for 2 h, during which a noticeable color change occurred. The temperature was then raised to 90°C, reducing the solution volume to ~20 mL (Figure 2B), resulting in the formation of a thick gel. This gel was then placed in an oven for heating, leading to the appearance of a brown precipitate. Once fully dried, the precipitate underwent calcination at 700°C for 2 h, ultimately yielding fine white ZnO powder (Figure 2C).

### 2.3. Synthesis and Characterization of ZnO NPs–Chsn

**Figure 3:**
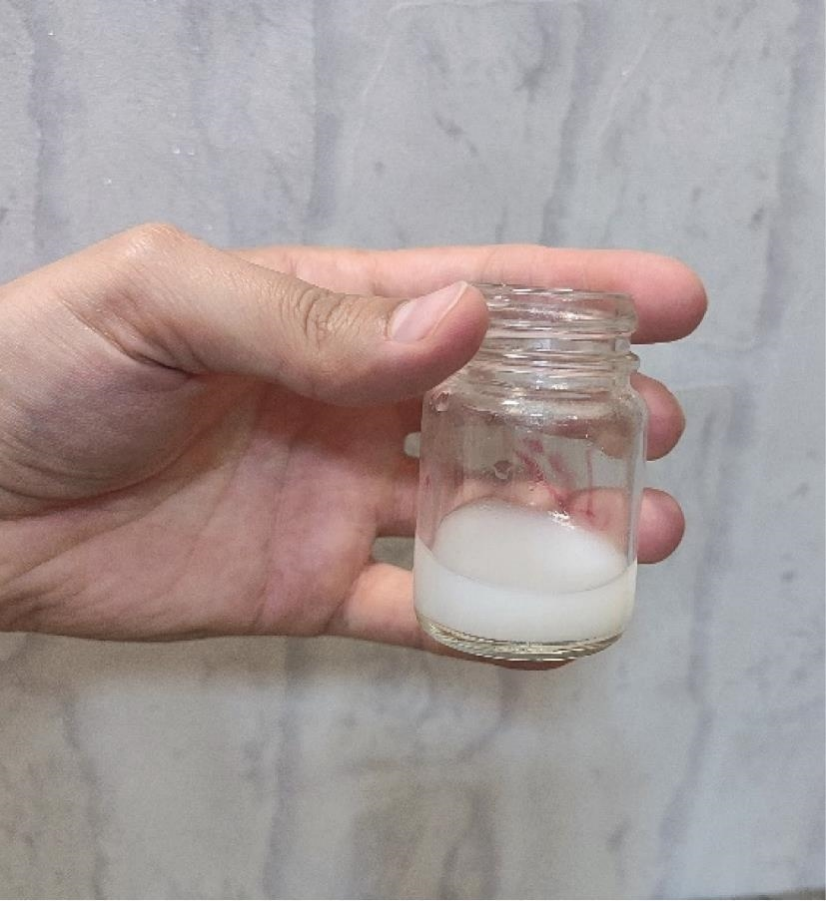
Homogeneous white suspension of green‐synthesized ZnO NPs dispersed in double‐distilled water after 20 min of ultrasonication.

To prepare a 1% acetic acid solution, 2.5 mL of acetic acid was diluted in 250 mL of double‐distilled water. Then, 0.5 g of Chsn powder was added to this solution and stirred continuously for 24 h to obtain a transparent 0.2% Chsn solution. Next, 0.25 g of ZnO NPs, synthesized through an eco‐friendly method, was dispersed in 20 mL of double‐distilled water using an ultrasonic device for 20 min to achieve a homogeneous white suspension (Figure 3). This suspension was then gradually introduced into the Chsn solution while maintaining constant stirring. Upon mixing, the solution’s color gradually shifted from white to colorless. The pH of the mixture was adjusted to 10 using a 2M NaOH solution, with continuous monitoring through a pH meter. At this pH level, the solution formed a stable milky colloid. The colloidal mixture was then left undisturbed for settling, followed by high‐speed centrifugation to separate the NPs. After centrifugation, the clear supernatant was carefully removed, and the remaining material was dried in an oven at 60°C, yielding the final product.

The characterization of the synthesized NPs was carried out using multiple analytical techniques. The morphology and structural characteristics were analyzed through transmission electron microscopy (TEM; Zeiss EM10C‐100 kV). Zeta potential analysis (HORIBA SZ‐100‐Z) was performed to evaluate surface charge and colloidal stability. Furthermore, scanning electron microscopy (SEM; SAMx and TESCAN BRNO‐Mira3 LMU) was employed to examine the surface morphology of the composite.

### 2.4. Preparation of Polyvinyl Alcohol/Sodium Alginate/Saffron (PSS)/ZnO NPs–Chsn Polymeric Hydrogel

**Figure 4:**
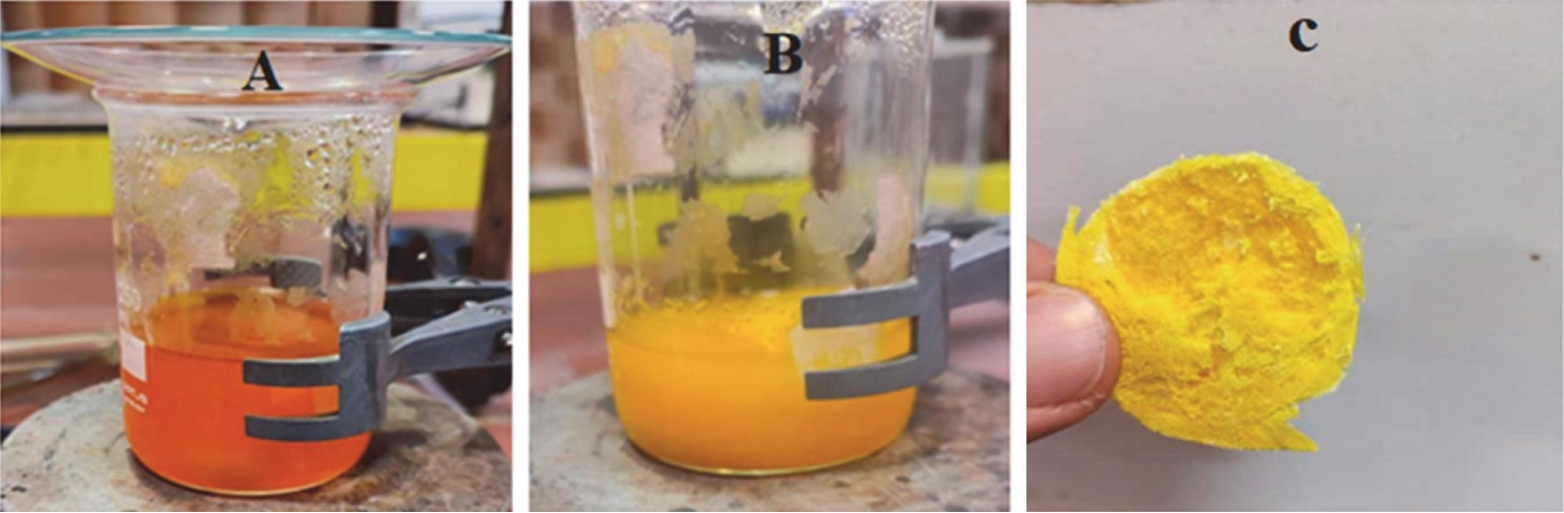
Preparation steps of the PSS/ZnO NPs–Chsn polymeric hydrogel. (A) Homogeneous mixture of polyvinyl alcohol (PVA), sodium alginate (SA), and saffron extract after stirring at room temperature. (B) Addition and dispersion of ZnO NPs–Chsn into the PSS solution with further stirring. (C) Final freeze‐dried hydrogel sheet after cross‐linking and freeze–thaw cycles, showing a stable porous structure.

The synthesis of the SA and PVA hydrogel begins with the preparation of a 10% (w/v) PVA solution by dissolving the required amount of PVA in deionized water under continuous stirring at 80°C until complete dissolution is achieved. The two solutions are then mixed in a 60:40 (v/v) ratio of PVA to SA and gently stirred overnight to ensure uniform blending. Subsequently, a certain amount of saffron extract (0.1% w/v) is dissolved in deionized water and added to the PVA/SA mixture, followed by continuous stirring for 1 h to ensure homogeneity (Figure 4A). Meanwhile, ZnO NPs–Chsn (1% w/v) are dispersed in water and gradually introduced into the PVA/SA/saffron (PSS) solution, followed by stirring for an additional 2 h to ensure proper distribution (Figure 4B). For cross‐linking, a 5% (w/v) calcium chloride (CaCl_2_) solution is added to the cooled hydrogel mixture in a petri dish, allowing it to react for 24 h. After cross‐linking, excess CaCl_2_ is removed, and the hydrogels are washed thoroughly three times with double‐distilled water to eliminate residual ions. To enhance structural stability, the hydrogel undergoes a freezing–thawing process, involving freezing at −20°C for 18 h followed by thawing at room temperature for 6 h, repeated for three cycles. Finally, the samples are subjected to freeze–drying for 24 h to obtain a porous, stable hydrogel (Figure 4C).

### 2.5. Hydrogel Characterization

#### 2.5.1. Swelling Ratio of Hydrogel

**Figure 5:**
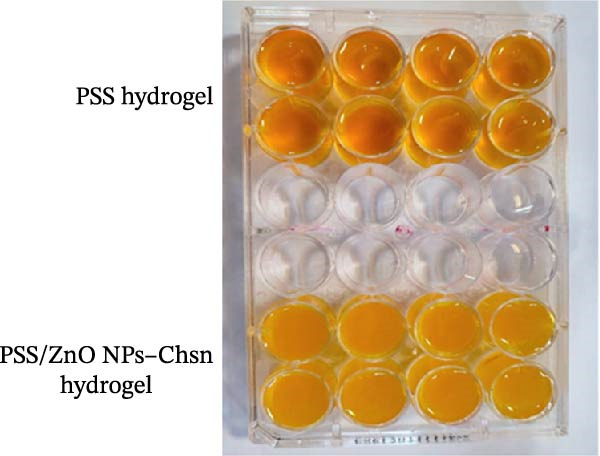
Swelling ratio assessment of PSS and PSS/ZnO NPs–Chsn hydrogels. The hydrogels were cast into a 24‐well plate and subjected to cross‐linking, freeze–thaw cycles, and freeze–drying. The top two rows contain PSS hydrogels, and the bottom two rows contain PSS/ZnO NPs–Chsn hydrogels, all with identical initial dry weights (*W*_*d*_) for accurate comparison.

The swelling properties of the hydrogels were analyzed using a gravimetric approach. To ensure accuracy in the evaluation, the synthesized hydrogel samples were first poured into 24‐well plates, followed by cross‐linking, freeze–thawing, and freeze–drying procedures (Figure 5).

After completing these steps, the samples were prepared for the experiment with identical initial dry weights (*W*_d_). Water absorption was measured through gravimetric analysis by recording the hydrogel’s mass overtime. Initially, the dry weight of the samples was determined. The hydrogels were then placed in a sieve, which was submerged in a beaker filled with water to facilitate swelling. At predetermined time intervals, the sieves were removed, and any excess surface water was carefully blotted using a paper towel. The samples were weighed before being returned to the water solution for further swelling. To ensure reliability, three separate specimens were tested. The swelling percentage (S%) was calculated using the following equation (Equation (1)) [28]:

(1) S%=Ww-Wd/Wd×100,

where *W*_w_ represents the weight of the swollen hydrogel, and *W*_*d*_ is the initial dry weight of the sample. The results were reported as the statistical mean along with the standard deviation.

#### 2.5.2. Hydrogel Porosity Measurements

The porosity of the hydrogels was assessed through the liquid displacement method. The freeze‐dried samples were fully immersed in ethanol until saturation was achieved, after which they were weighed again. This procedure was performed three times to ensure accuracy. The porosity of the hydrogels was then calculated using the following equation (Equation (2)) [29]:

(2) Porosity%=W2-W1/ρV×100.

Here, *W*_1_ and *W*_2_ represent the weights of the hydrogels before and after immersion in absolute ethanol, respectively. The parameter ρ denotes the density of absolute ethanol (0.785 g/cm^3^), while *V* refers to the volume of the sample prior to immersion.

#### 2.5.3. Morphological Examination

To investigate the porous nature of the hydrogels and to observe the distribution of ZnO NPs–Chsn within the pores, field emission SEM (FE‐SEM) was employed. The freeze‐dried hydrogel samples were carefully sectioned into 1 × 1 cm^2^ pieces and coated with a thin layer of gold to enhance conductivity.

Moreover, energy‐dispersive X‐ray spectroscopy (EDX) was performed in conjunction with FE‐SEM to identify the elemental composition of the hydrogels and assess how uniformly ZnO NPs–Chsn was distributed throughout the hydrogel matrix. This analysis provided insight into the material’s structural integrity and NPs dispersion.

#### 2.5.4. Contact Angle (CA) Measurement

The CA test was conducted to evaluate the wettability and liquid interaction properties of the hydrogel membranes. This technique measures the angle formed where a liquid droplet contacts the hydrogel surface, providing crucial insights into whether the material exhibits hydrophilic or hydrophobic behavior. For biomedical applications, particularly those involving direct skin contact, maintaining an appropriate balance between hydrophilicity and hydrophobicity is critical. Hydrogels with a CA measurement below 90° are classified as hydrophilic [30]. In this study, the CA of the freeze‐dried PSS/ZnO NPs–Chsn hydrogel (1 × 1 cm^2^) was determined using a CAG‐20 Jikan instrument. The recorded angles were processed and analyzed with Jika Assistant software (version 3.5). Additionally, when image noise was detected, further refinement and analysis were carried out using ImageJ software.

### 2.6. Evaluation of Antibacterial Activity

The antibacterial potential of the hydrogels was evaluated using the disc diffusion method. For this study, bacterial strains were obtained from the Iranian Research Organization for Science and Technology (IROST) in Tehran, Iran. The selected strains included the Gram‐positive bacteria *Staphylococcus aureus* (ATCC 25923) and *Bacillus cereus* (KCCM 40935), as well as the Gram‐negative strain *Escherichia coli* (ATCC 25922). A bacterial suspension with a concentration of 1.5 × 10^8^ CFU/mL was prepared as the inoculum. Nutrient Agar was used as the culture medium. It was dissolved in distilled water, autoclaved at 121°C for 15 min at 15 psi, and then cooled before 25 mL of the medium was poured into each petri dish. An inoculum suspension (0.1 mL) was uniformly spread onto the surface of the agar medium using a sterile L‐shaped glass rod to ensure even bacterial distribution. To achieve uniform growth, the plates were streaked with a sterile swab in one direction, rotated 90°, and streaked again. This procedure was repeated three times. The inoculated plates were then allowed to dry for approximately 5 min. Subsequently, two wells (6 mm in diameter) were aseptically created in each agar plate using a sterile corn borer. The wells were filled with PSS, PSS/Chsn, PSS/ZnO NPs, and PSS/ZnO NPs–Chsn polymeric hydrogels, whereas the standard antibiotic gentamicin was applied as a disc. The plates were incubated at 37°C for 24 h. Following incubation, the antibacterial efficacy was assessed by measuring the diameter of inhibition zones around each sample using Image J software.

### 2.7. MTT Assay

The cytocompatibility of PSS and PSS/ZnO NPs–Chsn hydrogels was evaluated using the MTT assay on primary human dermal fibroblast (HDF) cells. Cells were cultured in DMEM/F12 medium supplemented with 10% fetal bovine serum (FBS) and 1% penicillin–streptomycin, and seeded at a density of 5 × 10^3^ cells/well in 96‐well plates. Plates were incubated for 24 h at 37°C in a humidified atmosphere containing 5% CO_2_ to allow cell attachment. Subsequently, wells were treated with 10 μL of hydrogel extract and incubated for 24, 48, and 72 h. Phosphate‐buffered saline (PBS) was used as the negative control. For preparation of the hydrogel extract, sterilized hydrogel samples were immersed in serum‐free DMEM at a ratio of 0.2 g/mL and incubated at 37°C for 24 h. After incubation, the supernatant was collected and filtered through a 0.22 μm syringe filter to remove any residual particles, which was then used as the conditioned medium for cell treatment. Following treatment, cells were incubated with MTT solution (3‐(4,5‐dimethylthiazol‐2‐yl)‐2,5‐diphenyl tetrazolium bromide; Sigma, USA) at 37°C for 4 h. Subsequently, a 1% sodium dodecyl sulfate (SDS) solution was added to each well, and the plates were incubated for an additional 16 h at 37°C to solubilize the formazan crystals. The optical density (OD) was measured at 550 nm using a microplate reader.

(3) %Cell Viability=OD value of treated cells/OD value of untreated cellscontrol×100.

### 2.8. Statistical Analysis

We employed the SPSS software package (version 16.0, IBM, Chicago, IL, USA) to perform the statistical analysis. The values were analyzed by one‐way analysis of variance (ANOVA), followed by Duncan’s multiple range test (DMRT). All these results were expressed as mean ± SD for three independent experiments. *p*  < 0.05 was considered as statistically significant.

## 3. Results and Discussion

### 3.1. Characterization of NPs (ZnO NPs and ZnO NPs–Chsn)

#### 3.1.1. Morphological Analysis

**Figure 6:**
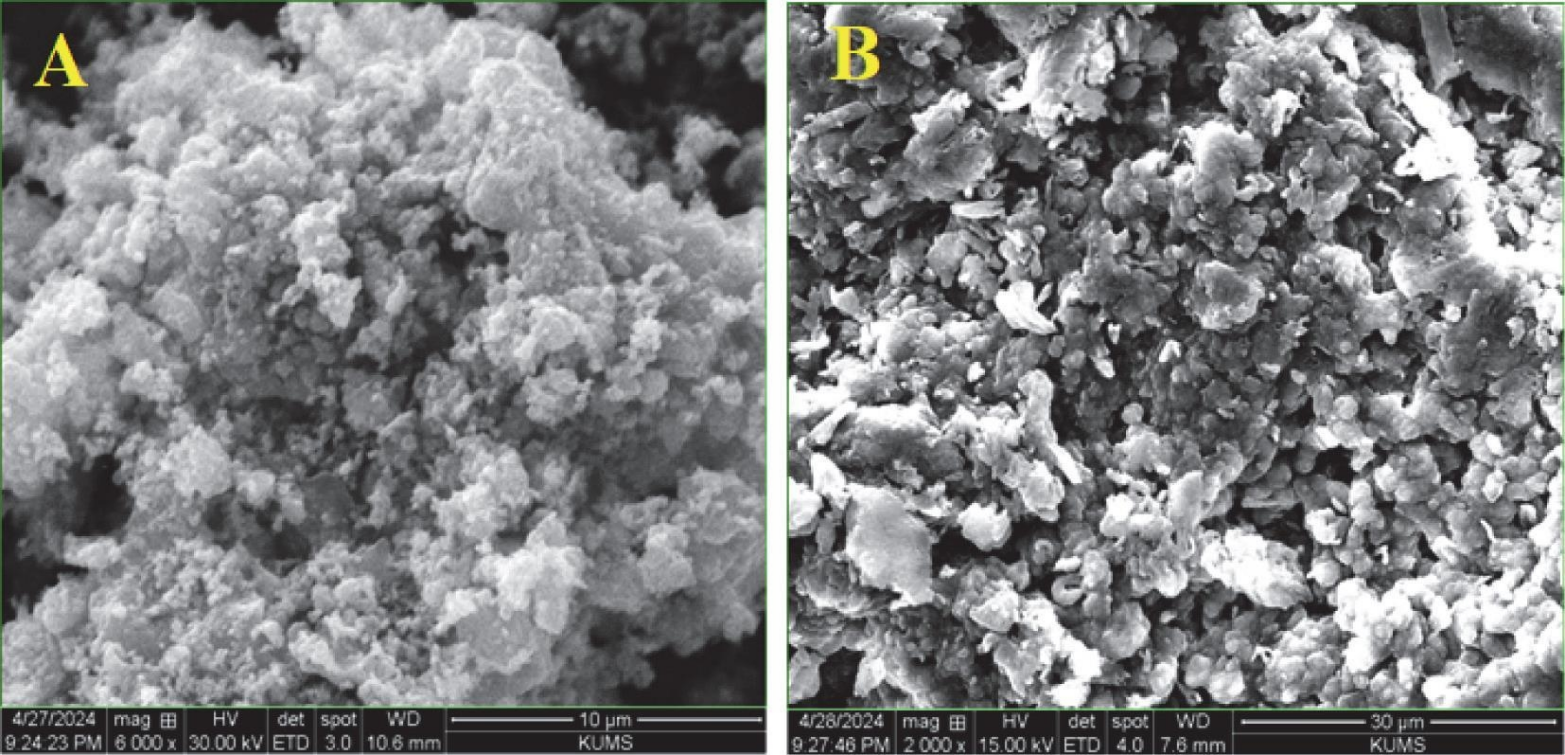
SEM images of (A) ZnO NPs and (B) ZnO NPs–Chsn.

The morphology of the green‐synthesized ZnO NPs in image (Figure 6A) exhibits an aggregated [31, 32], porous, and rough structure, primarily due to the presence of biological compounds in the quince peel extract, such as polyphenols, flavonoids, and other organic components. These bioactive compounds act as both stabilizing and reducing agents, facilitating Zn^2+^ ion reduction and NP nucleation. However, they also enhance hydrogen bonding and van der Waals interactions between NPs, leading to increased agglomeration and the formation of porous structures with high surface roughness. This aggregation can influence the overall surface area and reactivity of the NPs, which may affect their functional properties.

In the image (Figure 6B), after being coated with Chsn (ZnO NPs–Chsn), the NPs exhibit a more uniform and less dense structure with distinct shell‐like and sheet‐like morphologies [32]. Chsn, as a biocompatible stabilizing agent, significantly reduces interparticle forces and sterically hinders NP agglomeration, thereby enhancing their dispersion and colloidal stability. This surface modification not only alters the morphology but also improves the functional properties of the NPs, including increased biocompatibility, enhanced antibacterial efficacy, and potential for controlled drug delivery. The interaction between Chsn and ZnO may also influence surface charge, thereby impacting cellular interactions and bioactivity.

#### 3.1.2. Zeta Potential Analysis

**Figure 7:**
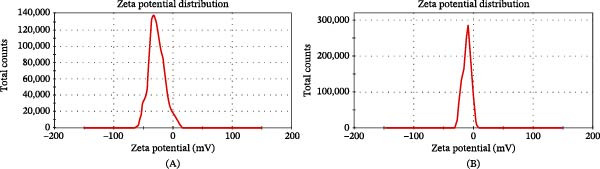
Zeta potential distribution of (A) ZnO NPs green synthesized using extract of quince fruits (*Cydonia oblonga*) and (B) ZnO NPs–Chsn.

Zeta potential is a key parameter for evaluating the colloidal stability of NPs, as it determines their surface charge and directly influences dispersion and aggregation behavior [33]. In this study, ZnO NPs were synthesized via a green synthesis approach and subsequently coated with Chsn. To investigate the impact of Chsn coating on the surface charge of the NPs, zeta potential measurements were performed for both samples, as shown in Figure 7A,B. The results indicate that green‐synthesized ZnO NPs (Figure 7A) exhibit a zeta potential of −27 mV. This relatively high negative value suggests good colloidal stability due to strong electrostatic repulsion between NPs, which helps prevent aggregation. The high negative charge can be attributed to the presence of biomolecules such as polyphenols, flavonoids, and other secondary metabolites from the plant extract used in the synthesis process [34]. After coating the ZnO NPs with Chsn, the zeta potential increased to −11.6 mV (Figure 7B), indicating a reduction in the negative surface charge. This shift can be explained by the cationic nature of Chsn, which, upon adsorption onto the NP surface, neutralizes some of the negative charges, bringing the zeta potential closer to zero. While this reduction in zeta potential might lower colloidal stability, the presence of Chsn introduces steric stabilization, which can counteract aggregation to some extent. Additionally, Chsn coating can improve the biocompatibility and functionality of the NPs by enhancing their interaction with biological systems and facilitating antimicrobial activity through membrane disruption and metabolic inhibition. Moreover, the NPs are moderately stable in aqueous solution, according to the negative signed value [35]. Overall, the observed changes in zeta potential confirm that Chsn coating significantly alters the surface properties of ZnO NPs. These modifications can influence their biological performance, cellular interactions, and potential applications in biomedical fields such as antibacterial treatments and controlled drug release.

#### 3.1.3. TEM Images

**Figure 8:**
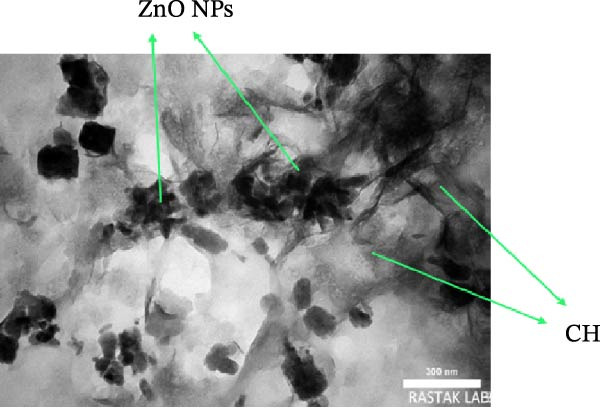
TEM image of ZnO NPs coated with Chsn, showing the morphology and distribution of ZnO NPs within the Chsn matrix.

The TEM images of ZnO NPs synthesized by the green method show NPs successfully coated within a Chsn polymer matrix (Figure 8). The morphology of the NPs is predominantly semi‐spherical, although some particles have irregular shapes. The darker areas in the image correspond to the ZnO NPs, which exhibit higher electron density, thus creating more contrast, while the lighter areas are attributed to the amorphous Chsn structure. The measurements indicate that the average size of the NPs is ~71.93 nm, which falls within the nanometric range and is ideal for biomedical and pharmaceutical applications. This reduction in size compared to other synthesis methods may be attributed to the role of organic compounds in the quince peel extract, which act as natural stabilizers and prevent excessive NP growth. Strong interactions between Chsn and ZnO NPs, likely due to hydrogen bonds and electrostatic interactions, have contributed to improved dispersion and stability of the nanocomposite. Moreover, the presence of Chsn during the coating phase enhances the stability and proper distribution of the NPs, leading to improved biological performance and antibacterial properties. These features make the ZnO NPs coated with Chsn suitable for applications in drug delivery, tissue engineering, and antibacterial properties [32].

### 3.2. Characterization of Hydrogels

#### 3.2.1. Investigation of Morphology and Structure of Hydrogels Using FE‐SEM

**Figure 9:**
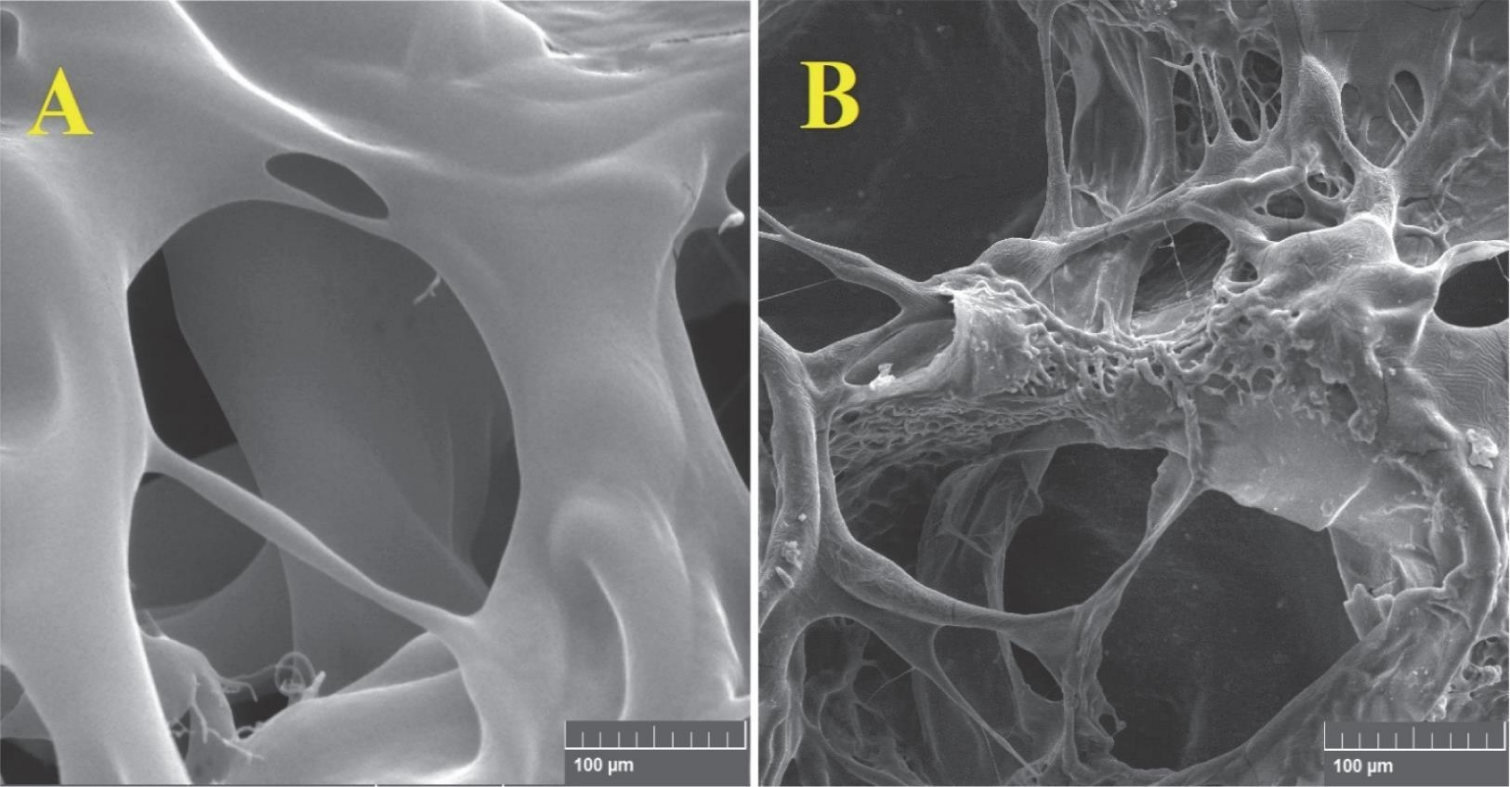
FE‐SEM images of hydrogel morphology: (A) microstructure of PSS hydrogel, exhibiting a highly porous and interconnected network with smooth pore walls and uniform polymer distribution and (B) PSS/ZnO NPs–Chsn hydrogel after incorporation of ZnO NPs–Chsn, showing a denser polymeric matrix with increased surface roughness and structural reinforcement, attributed to Chsn crosslinking and strong interactions between NPs and the hydrogel network.

The morphological characteristics of the synthesized hydrogels were investigated using FE‐SEM. Figure 9A represents the microstructure of the PSS hydrogel, while Figure 9B depicts the same hydrogel after the incorporation of ZnO NPs–Chsn.

**Figure 10:**
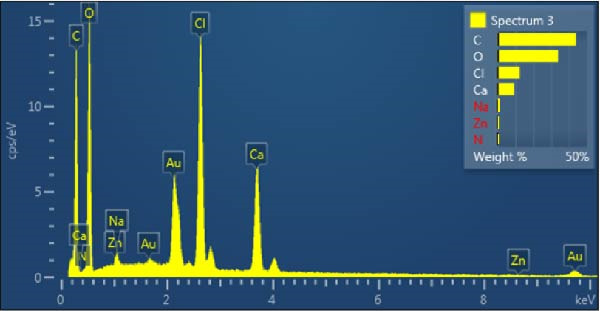
EDX analysis results for the PSS/ZnO NPs–Chsn hydrogel, demonstrating the elemental composition and the presence of ZnO NPs–Chsn in the PSS hydrogel structure.

In Figure 9A, the PSS/ZnO NPs–Chsn hydrogel exhibits a highly porous, interconnected network structure, which is a crucial feature for hydrogels, enabling efficient water retention, drug diffusion, and enhanced bioactivity [36]. The smooth and continuous pore walls suggest a homogeneous polymeric distribution, which ensures optimal swelling behavior and flexibility. The incorporation of saffron into the polymer matrix does not seem to significantly affect the pore morphology, but it may enhance the antibacterial and therapeutic properties of the hydrogel. In Figure 9B, following the incorporation of ZnO NPs–Chsn, notable structural modifications are observed. The hydrogel matrix appears denser, with reduced pore size and increased surface roughness. This transformation can be attributed to the crosslinking effect of Chsn, which enhances intermolecular interactions within the hydrogel network. The presence of ZnO NPs–Chsn leads to the formation of a compact and reinforced polymeric structure, which can significantly improve the hydrogel’s mechanical strength and stability [37]. Additionally, the rough and heterogeneous surface observed in Figure 9B suggests strong NP‐hydrogel interactions, which may contribute to improved cell adhesion, protein interactions, and antibacterial activity. Additionally, EDX analysis (Figure 10) acknowledged C, O, Na, Ca, Cl, N, and Zn in the PSS/ZnO NPs–Chsn hydrogel, which related to the PVA, SA polymers, saffron, and ZnO NPs–Chsn. The presence of Ca confirms the successful interaction between the polymeric chains and the cross‐linking agent [38]. The presence of Zn correlates with the incorporation of ZnO NPs–Chsn in the PSS hydrogel [39].

#### 3.2.2. Hydrogel Swelling Ratio

**Figure 11:**
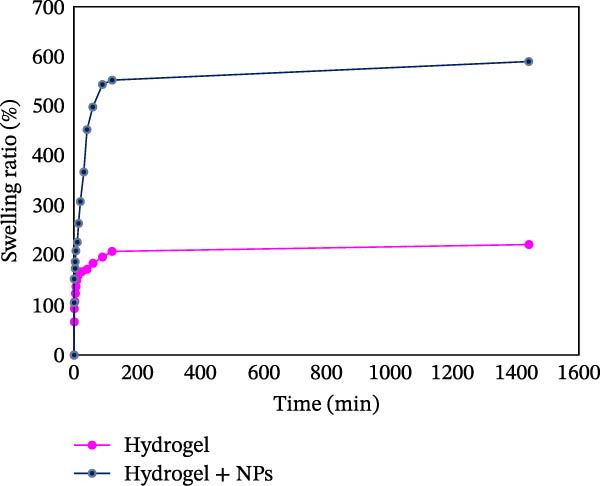
Swelling ratio (%) versus time (min) for the PSS hydrogel and the PSS/ZnO NPs–Chsn hydrogel. The incorporation of nanoparticles significantly enhances the water absorption capacity and swelling behavior.

An optimal polymer/metal nanocomposite hydrogel with antimicrobial properties should effectively interact with waterborne pathogens. Therefore, its swelling behavior is a crucial factor for its functionality and application [39].The presented graph illustrates the water absorption (swelling ratio) of two types of hydrogels (Figure 11): PSS hydrogel and the same hydrogel incorporated with ZnO NPs–Chsn, over a specific time period. The horizontal axis represents time (min), while the vertical axis shows the swelling ratio as a percentage. Analyzing this graph provides valuable insights into the swelling behavior and water absorption capacity of these materials, which are crucial for biomedical and tissue engineering applications. A comparison between the two samples reveals that the PSS hydrogel (pink curve) absorbs a certain amount of water during the initial minutes, reaching a swelling ratio of 220.91% ± 1.89%, after which it stabilizes with minimal further changes. In contrast, the hydrogel containing ZnO NPs–Chsn (dark blue curve) exhibits a much faster water absorption rate, rapidly reaching 589.39% ± 1.77% before leveling off. This significant difference indicates that the presence of NPs has a profound impact on both the swelling kinetics and the maximum water uptake capacity [28]. The enhanced water absorption in the NPs‐incorporated hydrogel is likely due to improved network structure and increased hydrophilicity. ZnO NPs–Chsn can modify the polymer matrix by creating a more porous structure, facilitating greater water retention within the hydrogel network. Additionally, Chsn itself is a hydrophilic polymer that enhances the hydrogel’s water absorption capacity. Moreover, ZnO NPs may contribute to stronger electrostatic interactions between polymer chains, further improving the swelling properties. Examining the stabilization of water absorption, the graph shows that both hydrogels reach a stable state after an initial period of rapid swelling. For PSS hydrogel (pink curve), the swelling ratio increases in the first 50–100 min, after which it stabilizes at 220.91% ± 1.89% and remains nearly unchanged for the rest of the experiment. This indicates that the hydrogel reaches its equilibrium swelling capacity relatively quickly and does not absorb significantly more water beyond this point. In contrast, the hydrogel with ZnO NPs–Chsn (dark blue curve) exhibits a much faster swelling rate, rapidly increasing within the first 50 min and reaching a plateau at 589.39% ± 1.77%. After this point, the swelling ratio shows only minor variations, indicating that the hydrogel has reached its maximum water absorption capacity. Thus, the equilibrium swelling state is reached much earlier for the NPs‐incorporated hydrogel compared to PSS hydrogel. The enhanced swelling rate in the modified hydrogel suggests that the NPs improve the water uptake dynamics, allowing the material to absorb and retain a significantly higher amount of water in a shorter period. The findings from this graph suggest that adding NPs to the hydrogel not only increases the maximum water absorption but also significantly enhances the swelling rate. This feature can be particularly beneficial in applications such as biomedicine, tissue engineering, drug delivery systems, and wound dressings, where controlling the rate and extent of fluid uptake is crucial for maintaining a moist environment and optimizing performance. Therefore, reinforcing hydrogels with NPs represents a promising strategy for improving their functional properties in biomedical applications.

#### 3.2.3. Hydrogel Porosity Measurements

The porosity measurement results showed that the porosity percentage of PSS hydrogel is 31.12% ± 1.03%, whereas, with the addition of the ZnO NPs–Chsn, this value increased significantly to 90.7% ± 1.83%. This remarkable increase in porosity can be attributed to the interactions between the NPs and the polymer matrix. The presence of ZnO NPs–Chsn disrupts the uniform arrangement of polymer chains, preventing excessive network density. Additionally, the functional groups in Chsn can form hydrogen bonds with polymer chains, leading to the formation of a 3D network with larger void spaces, thus increasing porosity. The enhanced porosity positively affects the antibacterial properties of the hydrogel. A more porous structure increases the surface area in contact with the environment, improving the antibacterial performance of ZnO NPs and Chsn. Moreover, higher porosity enhances the permeability of the hydrogel, facilitating the absorption and effective release of antibacterial agents. Furthermore, a porous environment can prevent bacterial aggregation and proliferation by creating unfavorable conditions for bacterial growth. Therefore, the increase in porosity not only improves the antibacterial performance of the hydrogel but also makes it a promising candidate for medical and disinfectant applications. The findings of the study by Kalantari et al. [28] align with the results obtained in our research, particularly in terms of the significant increase in porosity upon the addition of NPs to the hydrogel matrix. Their study demonstrated that incorporating CeO_2_ NPs (CeO_2_‐NPs) into Chsn/PVA hydrogels resulted in a highly porous structure (81%–90%), which was beneficial for wound healing applications [28]. This aligns well with our findings, where the incorporation of ZnO NPs–Chsn into PSS hydrogel increased porosity from 31.12% ± 1.03% to 90.7% ± 1.83%.

#### 3.2.4. CA Measurements

**Figure 12:**
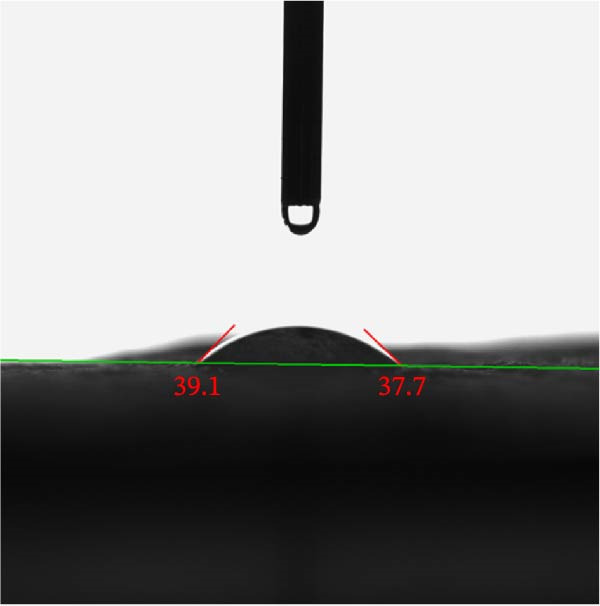
Contact angle (CA) analysis of PSS/ZnO NPs–Chsn hydrogel.

The CA of hydrogels is a key parameter for evaluating their surface wettability and hydrophilicity, which significantly influences their antibacterial performance. A smaller CA (below 90°) indicates a hydrophilic surface where water spreads more easily, promoting efficient interaction between the hydrogel and aqueous environments, such as infected wound exudates or body fluids. The average CA of 38.4° measured for the PSS/ZnO NPs–Chsn hydrogel (Figure 12) indicates a highly hydrophilic surface. This behavior can be attributed to the abundant hydrophilic functional groups present in PVA, SA, and Chsn, which facilitate water absorption and distribution across the hydrogel matrix. From an antibacterial perspective, high surface hydrophilicity enhances the diffusion and release of active antibacterial agents such as ZnO NPs–Chsn, increasing their availability at the infection site [40]. This promotes more effective bacterial contact, leading to improved antimicrobial action. Furthermore, the water‐attracting nature of the hydrogel can also help trap and immobilize bacteria, allowing localized antibacterial effects to be maximized. However, it is important to consider that excessive water uptake due to very low CA may compromise the mechanical integrity of the hydrogel or lead to unwanted swelling. Therefore, the observed moderate hydrophilicity (CA = 38.4°) in this study offers a favorable balance allowing sufficient fluid interaction and antibacterial agent mobility while maintaining structural stability. In summary, the measured CA demonstrates that the PSS/ZnO NPs–Chsn hydrogel possesses a surface suitable for enhanced antibacterial performance, making it a promising candidate for applications such as antimicrobial dressings, coatings, or delivery systems.

### 3.3. Antibacterial Assessments

**Figure 13:**
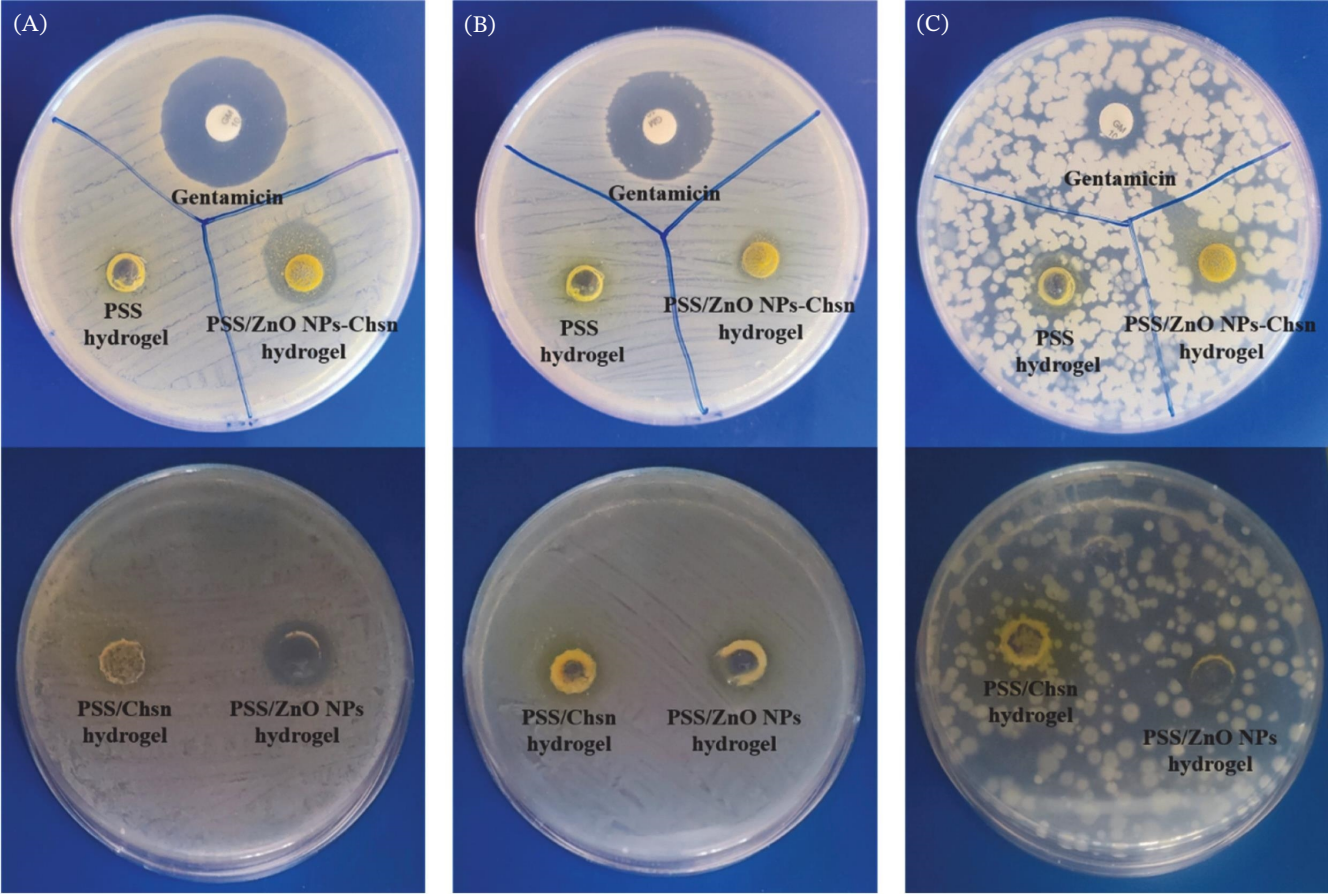
Antibacterial activity of PSS, PSS/Chsn, PSS/ZnO NPs, and PSS/ZnO NPs–Chsn polymeric hydrogels against (A) *Staphylococcus aureus*, (B) *Escherichia coli*, and (C) *Bacillus cereus* based on the agar diffusion method.

To assess the broad‐spectrum antimicrobial activity of PSS and PSS/ZnO NPs–Chsn hydrogels, tests were performed against three clinically relevant pathogenic microorganisms Gram‐positive bacteria *S. aureus* (ATCC 25923) and *B. cereus* (KCCM 40935), as well as the Gram‐negative strain *E. coli* (ATCC 25922). The disc diffusion experiment was employed for this evaluation. The results (Figure 13 and Table 1) showed that gentamicin, as the positive control, exhibited the highest inhibition against *S. aureus* (8.89 ± 0.04 mm) and *E. coli* (7.06 ± 0.06 mm), but its effect against *B. cereus* (2.44 ± 0.12 mm) was lower. This variation could be due to the intrinsic resistance of some Gram‐positive bacteria to gentamicin. The PSS hydrogel demonstrated very limited antibacterial activity, as it did not create a significant inhibition zone against *S. aureus* and *E. coli*, but showed a slight inhibition zone of 1.92 ± 0.19 mm against *B. cereus*. This finding suggests that the bioactive compounds present in saffron may exhibit antibacterial effects, although relatively weak. The incorporation of ZnO NPs–Chsn into the hydrogel significantly enhanced its antibacterial activity. Against *S. aureus*, the inhibition zone was 4.64 ± 0.20 mm, indicating improved efficacy compared to the base hydrogel. However, against *E. coli*, the ZnO NPs–Chsn hydrogel created only a 1.55 ± 0.10 mm inhibition zone, which was relatively smaller. This reduced effect may be due to the complex outer membrane of Gram‐negative bacteria, which can limit the penetration of antimicrobial agents [41]. Conversely, the highest antibacterial activity of the PSS/ZnO NPs–Chsn hydrogel was observed against *B. cereus*, with an inhibition zone of 4.94 ± 0.14 mm, even greater than that of gentamicin. To further clarify the contribution of each component, control hydrogels based on PSS containing either ZnO NPs or Chsn alone were evaluated. Against *E. coli*, both PSS/ZnO‐only and PSS/Chsn‐only hydrogels exhibited negligible antibacterial activity. For *S. aureus*, the PSS/Chsn‐only hydrogel was inactive, whereas the PSS/ZnO‐only hydrogel showed modest antibacterial effects, substantially lower than the PSS/ZnO NPs–Chsn hydrogel. In the case of *B. cereus*, PSS/Chsn‐only hydrogel remained ineffective, while PSS/ZnO‐only hydrogel demonstrated moderate antibacterial activity, again lower than the combined hydrogel. These results provide clear evidence of a synergistic interaction between ZnO NPs and Chsn, significantly enhancing the antibacterial efficacy of the PSS/ZnO NPs–Chsn hydrogel.

**Table 1:** Antibacterial results of PSS, PSS/Chsn, PSS/ZnO NPs, and PSS/ZnO NPs–Chsn polymeric hydrogels against *Staphylococcus aureus*, *Escherichia coli*, and *Bacillus cereus* based on the agar diffusion method.

Tested materials		Zone of inhibition (mm)		
		*S. aureus*	*E. coli*	*B. cereus*
Gentamicin		8.89 ± 0.04	7.06 ± 0.06	2.44 ± 0.12
PSS hydrogel		0.00 ± 0.00	0.00 ± 0.00	1.92 ± 0.19
PSS/Chsn hydrogel		0.00 ± 0.00	0.00 ± 0.00	0.00 ± 0.00
PSS/ZnO NPs hydrogel		1.98 ± 0.22	0.73 ± 0.15	2.15 ± 0.41
PSS/ZnO NPs–Chsn hydrogel		4.64 ± 0.20	1.55 ± 0.10	4.94 ± 0.14

*Note:* Values are given as mean ± SD of three repeats in each group.

The enhanced antibacterial activity observed in the PSS/ZnO NPs–Chsn hydrogel can be attributed to the combined effects of ZnO NPs and Chsn, which both possess intrinsic antimicrobial properties. ZnO NPs exert their antibacterial effects through multiple mechanisms, including the generation of ROS, which induce oxidative stress in bacterial cells, leading to membrane damage, protein oxidation, and DNA fragmentation. Additionally, ZnO NPs disrupt bacterial membranes by interacting with negatively charged cell walls, increasing permeability and ultimately causing cell lysis. Another important mechanism is the release of Zn^2+^ ions, which interfere with bacterial enzyme functions and disrupt metabolic pathways, further inhibiting bacterial growth [42]. Chsn, on the other hand, enhances antibacterial activity through electrostatic interactions, where its positively charged molecules bind to bacterial membranes, disrupt their integrity, and cause leakage of intracellular components. Moreover, Chsn can inhibit nutrient uptake by forming a barrier around bacterial cells, preventing the absorption of essential nutrients, leading to bacterial death [43]. The synergistic effect of ZnO NPs and Chsn enhances bacterial inhibition, as Chsn stabilizes ZnO NPs and promotes better interaction with bacterial cells, increasing overall antimicrobial efficacy. Although the saffron‐containing hydrogel without ZnO NPs–Chsn did not exhibit strong antibacterial activity, the inclusion of saffron in this formulation was not solely aimed at providing antimicrobial effects. Saffron contains antioxidant, antibacterial and anti‐inflammatory compounds such as crocin, safranal, and picrocrocin, which contribute to wound healing, inflammation reduction, and tissue regeneration [44, 45]. This suggests that beyond potential antibacterial properties, saffron enhances the biological performance of the hydrogel. Additionally, some studies suggest that bioactive compounds in saffron may interact with NPs to increase bacterial membrane permeability, potentially improving the efficacy of ZnO NPs–Chsn. Moreover, metal oxide NPs, such as ZnO, may exhibit cytotoxicity, and saffron could act as a biocompatibility enhancer, reducing NP toxicity while preserving antimicrobial efficacy. Based on these findings, it can be concluded that the combination of ZnO NPs–Chsn and saffron in the hydrogel significantly enhances its antibacterial activity, particularly against Gram‐positive bacteria. ZnO NPs and Chsn contribute to bacterial inhibition through multiple mechanisms, while saffron primarily supports biocompatibility, anti‐inflammatory properties, and potential synergistic effects. The PSS/ZnO NPs–Chsn hydrogel demonstrated superior antibacterial activity compared to the base hydrogel, especially against *S. aureus* and *B. cereus*, making it a promising candidate for antibacterial applications. However, further studies are required to fully understand its mechanisms of action, optimize its formulation, and evaluate its safety for biomedical applications.

No significant impact of PSS/ZnO NPs–Chsn hydrogel on the viability of *E. coli* can be caused by many reasons. This can be related to the cell wall of the membrane, as the Gram‐positive bacteria possess a single cytoplasmic membrane surrounded by a multilayered peptidoglycan polymer, forming a thick cell wall. In contrast, the Gram‐negative cell wall has a more complex architecture, consisting of two membranes, an outer membrane and an inner cytoplasmic membrane, separated by a thin peptidoglycan layer located in the periplasmic space [46]. Moreover, the interaction between ZnO NPs–Chsn and bacteria not only inhibits the growth of bacteria but also induces a generation of ROS, which leads to cell death [47]. It has been established that Gram‐positive bacteria are more sensitive to ROS than Gram‐negative bacteria [28]. It is suggested that, to improve the performance of the hydrogel against *E. coli*, cell membrane permeability could be enhanced using effective compounds, ROS generation and Zn^2+^ release increased by employing smaller ZnO NPs (10–30 nm) or doping with Ag or Cu at low concentrations, and the hydrogel structure optimized by reducing crosslinking density or introducing pH‐sensitive bonds for faster release of active agents in infected environments, which will be addressed in future studies.

### 3.4. MTT Cell Viability Assay

**Figure 14:**
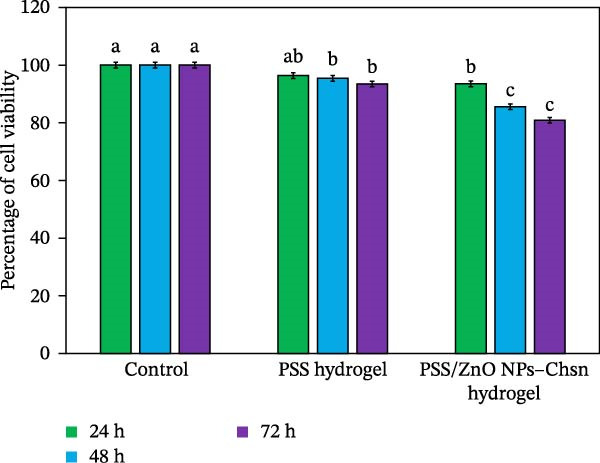
In vitro cytotoxicity assay on primary human dermal fibroblast (HDF) cells treated with PSS and PSS/ZnO NPs–Chsn hydrogels. Values are given as mean ± SD of three repeats in each group. Data are analyzed by one‐way ANOVA followed by Duncan’s multiple range tests. Column data marked with different letters indicate significant difference (*p* < 0.05).

The cytocompatibility of PSS and PSS/ZnO NPs–Chsn hydrogels was evaluated using the MTT assay on primary HDF cells. As shown in Figure 14, the PSS hydrogel maintained high cell viability (>93%) throughout the 72 h incubation period, whereas the PSS/ZnO NPs–Chsn hydrogel exhibited cell viabilities of 93.65% ± 1.81%, 85.64% ± 0.98%, and 81.05% ± 2.42% at 24, 48, and 72 h, respectively. Although a slight decrease in viability was observed with prolonged exposure, the values remained above the 80% threshold, indicating acceptable biocompatibility [38]. The superior cytocompatibility of the hydrogels can be attributed to the green and biocompatible nature of the PSS matrix, which may reduce the potential cytotoxicity of ZnO NPs. PBS was used as the negative control. Overall, the results suggest that PSS/ZnO NPs–Chsn hydrogels exhibit satisfactory cytocompatibility with primary HDF cells and, therefore, may be considered a promising candidate for further investigation in biomedical applications.

## 4. Conclusion

This study reports the development of a novel antibacterial hydrogel based on PSS, incorporating ZnO NPs that were greenly synthesized using reductive metabolites from quince (*Cydonia oblonga*) peel extract and subsequently coated with chitosan (Chsn) to enhance stability, biocompatibility, and antimicrobial activity. Incorporation of ZnO NPs–Chsn into the PSS hydrogel matrix resulted in a nanocomposite hydrogel (PSS/ZnO NPs–Chsn). Morphological, physicochemical, and antibacterial analyses demonstrated that ZnO NPs–Chsn significantly improved the porous structure, swelling behavior, and antibacterial efficacy of the hydrogel. Bacterial culture analyses revealed strong antibacterial activity, particularly against Gram‐positive strains such as *S. aureus* and *B. cereus*, highlighting its potential for biomedical applications. However, its performance against Gram‐negative bacteria, such as *E. coli*, was relatively weak. Future studies will focus on enhancing efficacy against Gram‐negative strains through optimization of NP size, doping with Ag or Cu, and modification of the hydrogel network for controlled release of active compounds. Further investigations will also aim to optimize structural and functional properties of the hydrogel to fully exploit its biomedical potential and improve wound‐healing capabilities.

## Funding

No funding was received for this research.

## Conflicts of Interest

The authors declare no conflicts of interest.

## Data Availability

The data that support the findings of this study are available from the corresponding author upon reasonable request.
